# Supplementary material for: How antisense transcripts can evolve to encode novel proteins
Source: Nat Commun. 2024 Jul 23;15:6187. doi: 10.1038/s41467-024-50550-3 (PMC11266595; doi:10.1038/s41467-024-50550-3)
Supplement: Supplementary file 1 — Supplementary Information [file 41467_2024_50550_MOESM1_ESM.pdf]

How antisense transcripts can evolve to encode novel proteins
Supplementary Material

Bharat Ravi Iyengar[1,†], Anna Grandchamp[1], Erich Bornberg-Bauer[1,2]

[1]Institute for Evolution and Biodiversity, University of Münster, Hüfferstrasse 1, 48149 Münster, Germany

[2]Department of Protein Evolution, Max Planck Institute for Biology Tübingen, Max-Planck-Ring 5, 72076 Tübingen, Germany

[†] Corresponding author: b.ravi@uni-muenster.de

Contents

1 Mutation rate and mutation rate bias in in *Drosophila melanogaster* **2**

2 Probability of asORFs in frames 0 and 2 is identical to that of igORFs of same length and GC-content **2**

3 Why asORFs appear to be most probable in frame 0, in *D. melanogaster* but not in *S. cerevisiae* **3**

4 Distribution of antisense ORFs in *Drosophila melanogaster* genome **5**

5 Translational efficiency of asORFs **6**

6 Gain and loss probabilities of antisense ORFs in *Drosophila melanogaster* **8**
- 6.1 Model predictions . . . 8
- 6.2 Analysis of asORF gain and loss using genomics data . . . 9

7 Effect of mutations on asORFs **11**

8 Is GC-content a better parameter for asORF probability calculation than global DNA oligomer frequencies? **14**

9 Information on the analysis scripts **16**
- 9.1 Modeling and general analysis . . . 16
- 9.2 Analysis of *S. cerevisiae* data . . . 16
- 9.3 Analysis of *D. melanogaster* data . . . 17

1. Mutation rate and mutation rate bias in in *Drosophila melanogaster*

**Substitution**	Probability(μ)
A:T→T:A	0.056
A:T→G:C	0.243
A:T→C:G	0.074
G:C→A:T	0.483
G:C→T:A	0.075
G:C→C:G	0.069

Table S1: Mutation bias probabilities for different nucleotide mutations based on Schrider *et al.* (2013) and Zhang and Gerstein (2003). A:T denotes an A-T base pair in a double stranded DNA. Thus A→G mutation on one DNA strand would cause a T→C mutation on the complementary strand. We describe the other mutations in the same way. We used an average mutation rate of 7.8×10^{-9} mutations per nucleotide position per generation (Schrider *et al.*, 2013)

2. Probability of asORFs in frames 0 and 2 is identical to that of igORFs of same length and GC-content

The probability of finding an antisense stop codon in frame 0 is same as the probability of finding the three reverse complementary codons in the sense ORF (TTA, CTA and TCA). These three codons are allowed in the sense ORFs, and their probability would be simply determined by the GC-content of the sense ORF. These three codons have the same GC composition as the stop codons, and therefore, their probability is identical to that of stop codons (given identical GC-content of the locus). Therefore, given these considerations, the probability of a frame-0 antisense ORF (asORF) is identical to that of an intergenic ORF (igORF) of same length and GC-content.

Next, we explain why the probability of frame-2 asORFs is identical to that of igORFs of similar nucleotide composition and length. The probability of finding a frame-2 antisense stop codon is determined by the corresponding dicodons in the sense ORF. There are 64 possible overlapping dicodons for any antisense codon in either frame 1 or frame 2 ($4^3 = 64$) because three out of six positions in a dicodon are determined by the overlapping antisense codon. Thus, there are $64 \times 3 = 192$ dicodons that overlap with any of the three antisense stop codons. By definition, the sense ORF should not contain a stop codon which means that no dicodon can contain a stop codon. For frame-1 antisense stop codons, 64 overlapping sense overlapping dicodons contain a stop codon (Table S2A), whereas for frame-2 antisense stop codons none of the overlapping dicodons contain a stop codon (Table S2B). Therefore, the probability

(A)

TAA				TAG				TGA			
AAT **TAA**	AAT TAT	AAT **TAG**	AAT TAC	AAC **TAA**	AAC TAT	AAC **TAG**	AAC TAC	AAT CAA	AAT CAT	AAT CAG	AAT CAC
TAT **TAA**	TAT TAT	TAT **TAG**	TAT TAC	TAC **TAA**	TAC TAT	TAC **TAG**	TAC TAC	TAT CAA	TAT CAT	TAT CAG	TAT CAC
GAT **TAA**	GAT TAT	GAT **TAG**	GAT TAC	GAC **TAA**	GAC TAT	GAC **TAG**	GAC TAC	GAT CAA	GAT CAT	GAT CAG	GAT CAC
CAT **TAA**	CAT TAT	CAT **TAG**	CAT TAC	CAC **TAA**	CAC TAT	CAC **TAG**	CAC TAC	CAT CAA	CAT CAT	CAT CAG	CAT CAC
ATT **TAA**	ATT TAT	ATT **TAG**	ATT TAC	ATC **TAA**	ATC TAT	ATC **TAG**	ATC TAC	ATT CAA	ATT CAT	ATT CAG	ATT CAC
TTT **TAA**	TTT TAT	TTT **TAG**	TTT TAC	TTC **TAA**	TTC TAT	TTC **TAG**	TTC TAC	TTT CAA	TTT CAT	TTT CAG	TTT CAC
GTT **TAA**	GTT TAT	GTT **TAG**	GTT TAC	GTC **TAA**	GTC TAT	GTC **TAG**	GTC TAC	GTT CAA	GTT CAT	GTT CAG	GTT CAC
CTT **TAA**	CTT TAT	CTT **TAG**	CTT TAC	CTC **TAA**	CTC TAT	CTC **TAG**	CTC TAC	CTT CAA	CTT CAT	CTT CAG	CTT CAC
AGT **TAA**	AGT TAT	AGT **TAG**	AGT TAC	AGC **TAA**	AGC TAT	AGC **TAG**	AGC TAC	AGT CAA	AGT CAT	AGT CAG	AGT CAC
TGT **TAA**	TGT TAT	TGT **TAG**	TGT TAC	TGC **TAA**	TGC TAT	TGC **TAG**	TGC TAC	TGT CAA	TGT CAT	TGT CAG	TGT CAC
GGT **TAA**	GGT TAT	GGT **TAG**	GGT TAC	GGC **TAA**	GGC TAT	GGC **TAG**	GGC TAC	GGT CAA	GGT CAT	GGT CAG	GGT CAC
CGT **TAA**	CGT TAT	CGT **TAG**	CGT TAC	CGC **TAA**	CGC TAT	CGC **TAG**	CGC TAC	CGT CAA	CGT CAT	CGT CAG	CGT CAC
ACT **TAA**	ACT TAT	ACT **TAG**	ACT TAC	ACC **TAA**	ACC TAT	ACC **TAG**	ACC TAC	ACT CAA	ACT CAT	ACT CAG	ACT CAC
TCT **TAA**	TCT TAT	TCT **TAG**	TCT TAC	TCC **TAA**	TCC TAT	TCC **TAG**	TCC TAC	TCT CAA	TCT CAT	TCT CAG	TCT CAC
GCT **TAA**	GCT TAT	GCT **TAG**	GCT TAC	GCC **TAA**	GCC TAT	GCC **TAG**	GCC TAC	GCT CAA	GCT CAT	GCT CAG	GCT CAC
CCT **TAA**	CCT TAT	CCT **TAG**	CCT TAC	CCC **TAA**	CCC TAT	CCC **TAG**	CCC TAC	CCT CAA	CCT CAT	CCT CAG	CCT CAC

(B)

TAA				TAG				TGA			
ATT AAA	ATT AAT	ATT AAG	ATT AAC	ACT AAA	ACT AAT	ACT AAG	ACT AAC	ATC AAA	ATC AAT	ATC AAG	ATC AAC
TTT AAA	TTT AAT	TTT AAG	TTT AAC	TCT AAA	TCT AAT	TCT AAG	TCT AAC	TTC AAA	TTC AAT	TTC AAG	TTC AAC
GTT AAA	GTT AAT	GTT AAG	GTT AAC	GCT AAA	GCT AAT	GCT AAG	GCT AAC	GTC AAA	GTC AAT	GTC AAG	GTC AAC
CTT AAA	CTT AAT	CTT AAG	CTT AAC	CCT AAA	CCT AAT	CCT AAG	CCT AAC	CTC AAA	CTC AAT	CTC AAG	CTC AAC
ATT ATA	ATT ATT	ATT ATG	ATT ATC	ACT ATA	ACT ATT	ACT ATG	ACT ATC	ATC ATA	ATC ATT	ATC ATG	ATC ATC
TTT ATA	TTT ATT	TTT ATG	TTT ATC	TCT ATA	TCT ATT	TCT ATG	TCT ATC	TTC ATA	TTC ATT	TTC ATG	TTC ATC
GTT ATA	GTT ATT	GTT ATG	GTT ATC	GCT ATA	GCT ATT	GCT ATG	GCT ATC	GTC ATA	GTC ATT	GTC ATG	GTC ATC
CTT ATA	CTT ATT	CTT ATG	CTT ATC	CCT ATA	CCT ATT	CCT ATG	CCT ATC	CTC ATA	CTC ATT	CTC ATG	CTC ATC
ATT AGA	ATT AGT	ATT AGG	ATT AGC	ACT AGA	ACT AGT	ACT AGG	ACT AGC	ATC AGA	ATC AGT	ATC AGG	ATC AGC
TTT AGA	TTT AGT	TTT AGG	TTT AGC	TCT AGA	TCT AGT	TCT AGG	TCT AGC	TTC AGA	TTC AGT	TTC AGG	TTC AGC
GTT AGA	GTT AGT	GTT AGG	GTT AGC	GCT AGA	GCT AGT	GCT AGG	GCT AGC	GTC AGA	GTC AGT	GTC AGG	GTC AGC
CTT AGA	CTT AGT	CTT AGG	CTT AGC	CCT AGA	CCT AGT	CCT AGG	CCT AGC	CTC AGA	CTC AGT	CTC AGG	CTC AGC
ATT ACA	ATT ACT	ATT ACG	ATT ACC	ACT ACA	ACT ACT	ACT ACG	ACT ACC	ATC ACA	ATC ACT	ATC ACG	ATC ACC
TTT ACA	TTT ACT	TTT ACG	TTT ACC	TCT ACA	TCT ACT	TCT ACG	TCT ACC	TTC ACA	TTC ACT	TTC ACG	TTC ACC
GTT ACA	GTT ACT	GTT ACG	GTT ACC	GCT ACA	GCT ACT	GCT ACG	GCT ACC	GTC ACA	GTC ACT	GTC ACG	GTC ACC
CTT ACA	CTT ACT	CTT ACG	CTT ACC	CCT ACA	CCT ACT	CCT ACG	CCT ACC	CTC ACA	CTC ACT	CTC ACG	CTC ACC

Table S2: The 192 sense dicodons overlapping an antisense stop codon in the antisense frames **(A)** 1 and **(B)** 2. We have highlighted the reverse complementary sequence corresponding to an antisense stop codon, in red font. In panel **(A)** the stop codon within 64 dicodons are highlighted with bold font.

of an antisense frame-2 stop codon is identical to that of a stop codon in an intergenic locus with identical GC-content.

3. Why asORFs appear to be most probable in frame 0, in *D. melanogaster* but not in *S. cerevisiae*

We analysed the differences between the predictions from the two species more closely. The most salient difference exists in the probability of asORFs in frame 0. The reason is that stop codons in frame 0 are 2.7 times more likely in *S. cerevisiae* than in *D. melanogaster* (Table S3). Therefore we analysed the frequency of these codons and their specific usage to encode the corresponding amino acids.

Stop codons in frame 0 overlap with the codons – TTA, CTA (coding for leucine) and TCA (coding for serine). Both leucine and serine are encoded by six codons. We analysed the coding regions of *S. cerevisiae* and *D. melanogaster* to estimate the codon usage for leucine and serine in both these organisms. We found that the total frequencies of leucine and serine are similar betwen the two organisms. However, the codons that overlap with an antisense stop codon are more frequently used in *S. cerevisiae* than in *D. melanogaster* (Figure S1).

	S. cerevisiae	*D. melanogaster*
Start codon	0.0169	0.0172
Stop codon: Frame 0	**0.0592**	**0.0216**
Stop codon: Frame 1	0.0399	0.0319
Stop codon: Frame 2	0.0482	0.0423

Table S3: Probability of start and stop codons in the three different antisense frames, calculated using distribution of codons and dicodons in *S. cerevisiae* and *D. melanogaster* coding sequences.

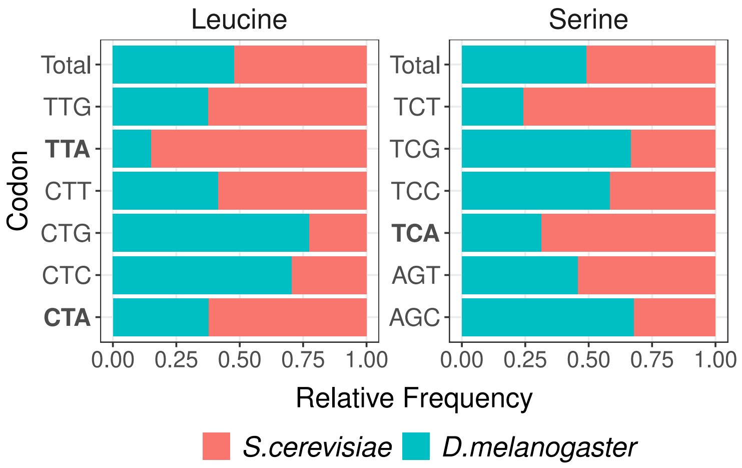

Figure S1: Codon usage of leucine and serine in *S. cerevisiae* and *D. melanogaster*. Codons highlighted in bold overlap with an antisense stop codon.

4. Distribution of antisense ORFs in *Drosophila melanogaster* genome

We identified antisense ORFs and intergenic ORFs using genome and transcriptome data from seven *D. melanogaster* lines (Grandchamp *et al.*, 2023). We performed the same analysis for every *D. melanogaster* line. Specifically, we first obtained the genome assembly, genome annotations and transcriptome assembly for each line (Grandchamp *et al.*, 2023). Next, we identified RNAs that overlap in antisense to any annotated protein coding gene. Next, we extracted ORFs in these antisense RNAs using *getorf* (Rice *et al.*, 2000), and mapped the genomic coordinates of these ORFs using nucleotide BLAST (100% query coverage and sequence identity; Altschul *et al.*, 1990; Camacho *et al.*, 2009). We restricted our further analyses to asORFs that completely overlap with an exon of a known protein coding genes, and themselves do not contain any intron. We did so because overlaps interrupted by introns (on both sense and antisense ORFs) can cause the different exons to overlap in different antisense frames. Thus one specific frame cannot be attributed to these asORFs. We thus identified asORFs in each *D. melanogaster* line, such that our search space for asORFs consists of all known protein coding exons, that are 100% overlapped with an antisense RNA (Table S4). We calculate the total antisense loci from this search space using Equations 4 – 6 (Main Text).

	**Denmark**	**Finland**	**Spain**	**Sweden**	**Türkiye**	**Ukraine**	**Zambia**
Total antisense loci	1440501	1272815	1326269	1119960	1641755	1143721	1200121
Expected asORF0	314 (327)	276 (290)	291 (304)	250 (256)	361 (374)	252 (262)	269 (279)
Observed asORF0	276	144	253	178	194	179	175
Expected asORF1	371 (299)	325 (265)	343 (279)	296 (230)	428 (338)	297 (239)	319 (252)
Observed asORF1	483	300	391	391	469	377	430
Expected asORF2	397 (327)	348 (290)	367 (304)	318 (256)	459 (374)	318 (262)	342 (279)
Observed asORF2	251	150	179	201	181	138	226
Total intergenic loci	2147483647	2147483647	2147483647	2147483647	2147483647	2147483647	2147483647
Expected igORF	1707687 (1768465)	1776758 (1839181)	1840872 (1906004)	1761983 (1823809)	1808499 (1873396)	1760268 (1822705)	1690669 (1750696)
Observed igORF	1763975	1828152	1889493	1807274	1858731	1811161	1740461

Table S4: Summary of antisense and intergenic ORFs identified in *D. melanogaster* lines. Expected numbers of ORFs within parantheses were estimated using GC-content of each locus, whereas those outside the parantheses were estimated using DNA oligomer frequencies. The different asORFs reported here include sub-ORFs within longer ORFs detected by *getorf*. Here we only report asORFs that do not contain introns and that completely overlap with a protein coding exon (sense ORF).

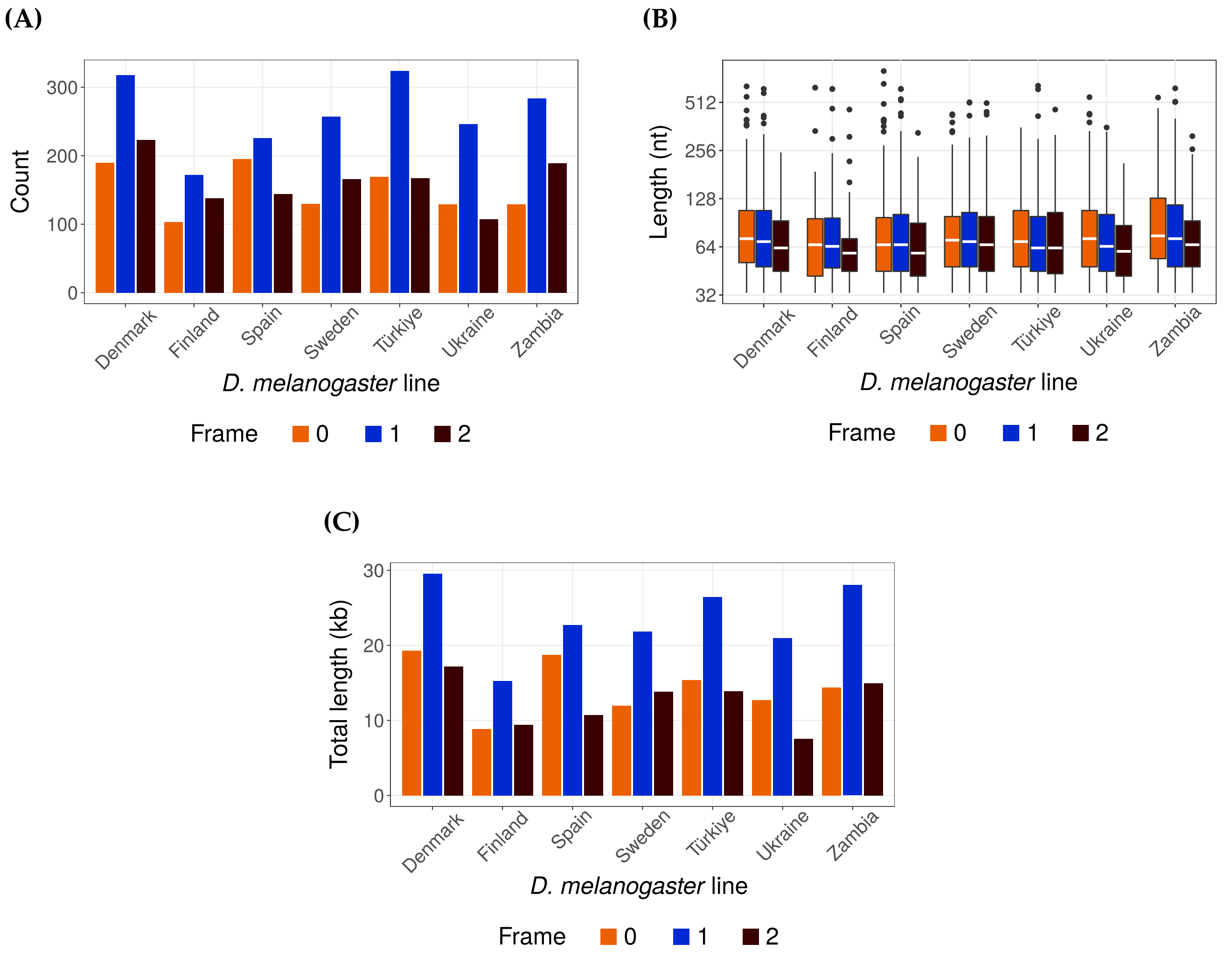

Figure S2: Properties of asORFs identified in the seven *D. melanogaster* lines. **(A)** Total number of asORFs (vertical axis). **(B)** asORF length distribution (vertical axis) denoted by boxplots where the boxes extend from the first to the third quartile and the whiskers have a length equal to 1.5 $\times$ the interquartile range. We indicate the median length using a white horizontal bar. **(C)** Cumulative length of all asORFs (vertical axis). In all the panels, the horizontal axes denote the seven different lines, and the bar colors denote the three different frames (0: orange, 1: blue, and 2: brown). We only show asORFs that overlap 100% with a previously annotated protein coding exon (Table S4).

5. Translational efficiency of asORFs

To estimate the translational efficiency of asORFs in *S. cerevisiae*, we used data from a recently published study (Wacholder *et al.*, 2023). This large dataset (iRibo) has been compiled from different published ribo-seq (sequencing of ribosomal footprint) experiments in *S. cerevisiae* such that every ORF (predicted or annotated) is assigned a number of reads that are in-frame with the ribosome's elongation periodicity. For every antisense-ORFs (as annotated by this study), we extracted the number of reads, and calculated the frame of overlap. We note that iRibo dataset is recent and was not available when we started our study. However, our analysis of asORFs from iRibo agrees with our model's predictions, and qualitatively agrees with the observed frequencies of asORFs shown in Table 2 and Figure 2 (Figure S3A/B). More specif-

ically, the asORFs in frame 1 are significantly more numerous than those in the other two frames (Figure S3A; one tailed Fisher exact test, FDR corrected $P < 10^{-22}$). The asORFs in frame 1 are also significantly longer than those in the other two frames (Figure S3B; one tailed Mann-Whitney U test, FDR corrected $P < 10^{-22}$). Next, we analysed if asORFs in frame 1 have more riboseq reads than those in the other two frames. We found that asORFs in frame 1 have significantly more reads than asORFs in frame 0 (one tailed Mann-Whitney U test, FDR corrected $P = 7.5 \times 10^{-3}$) but not asORFs in frame 2 (one tailed Mann-Whitney U test, FDR corrected $P = 0.115$). This does not indicate that there is no significant difference in the total translational output for asORFs in the different frames. That is so because both the number of asORFs and the translational efficiency is responsible for translational output. We found that the total translational output is significantly higher for asORFs in frame 1 than those in the other two frames (Figure S3A; one tailed Fisher exact test, FDR corrected $P < 10^{-22}$). Next, we compared the number of riboseq reads of the different asORFs and igORFs. We found that igORFs had a significantly larger number of reads than all asORFs (Figure S3B; one tailed Fisher exact test, FDR corrected $P < 10^{-22}$). More interestingly, the riboseq read count distribution of igORFs was bimodal. Specifically, a subset of igORFs was expressed more than the other subset, by two orders of magnitude. Interestingly, the length distribution of igORFs was also bimodal. These observations suggest that there are two different kinds of igORFs. The longer and highly translated igORFs could have undergone adaptive evolution.

To perform an analogous analysis for *D. melanogaster* asORFs, we did not find a compiled resource like iRibo. Therefore we used Kozak consensus sequence (KCS) score (Acevedo *et al.*, 2018) and ORF position in the RNA as proxies of translational efficiency as shown in another study (Patraquim *et al.*, 2022). We did not find any statistically significant difference between the values of these parameters for the different frames, that is also consistent across the seven different *D. melanogaster* lines (Mann-Whitney U test, 95% confidence interval, FDR corrected). We also did not find any significant difference between the KCS scores of igORFs and any of the three kinds of asORFs (Mann-Whitney U test, 95% confidence interval, FDR corrected).

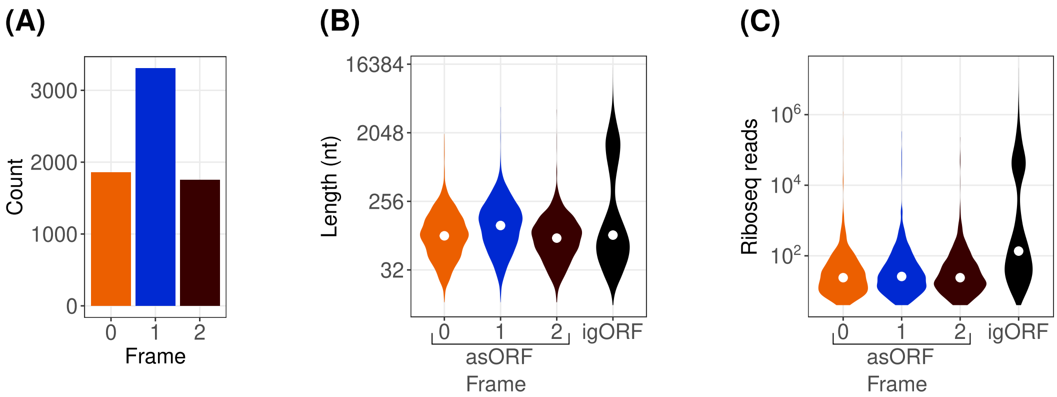

Figure S3: Yeast asORFs from iRibo (Wacholder *et al.*, 2023). Number of total asORFs (**A**, vertical axis), ORF length distribution of asORFs and igORFs (**B**, vertical axis), and riboseq reads distribution of asORFs and igORFs (**C** vertical axis), in each of the three frames (horizontal axis). We only show asORFs that overlap 100% with the sense ORF.

6. Gain and loss probabilities of antisense ORFs in *Drosophila melanogaster*

6.1 Model predictions

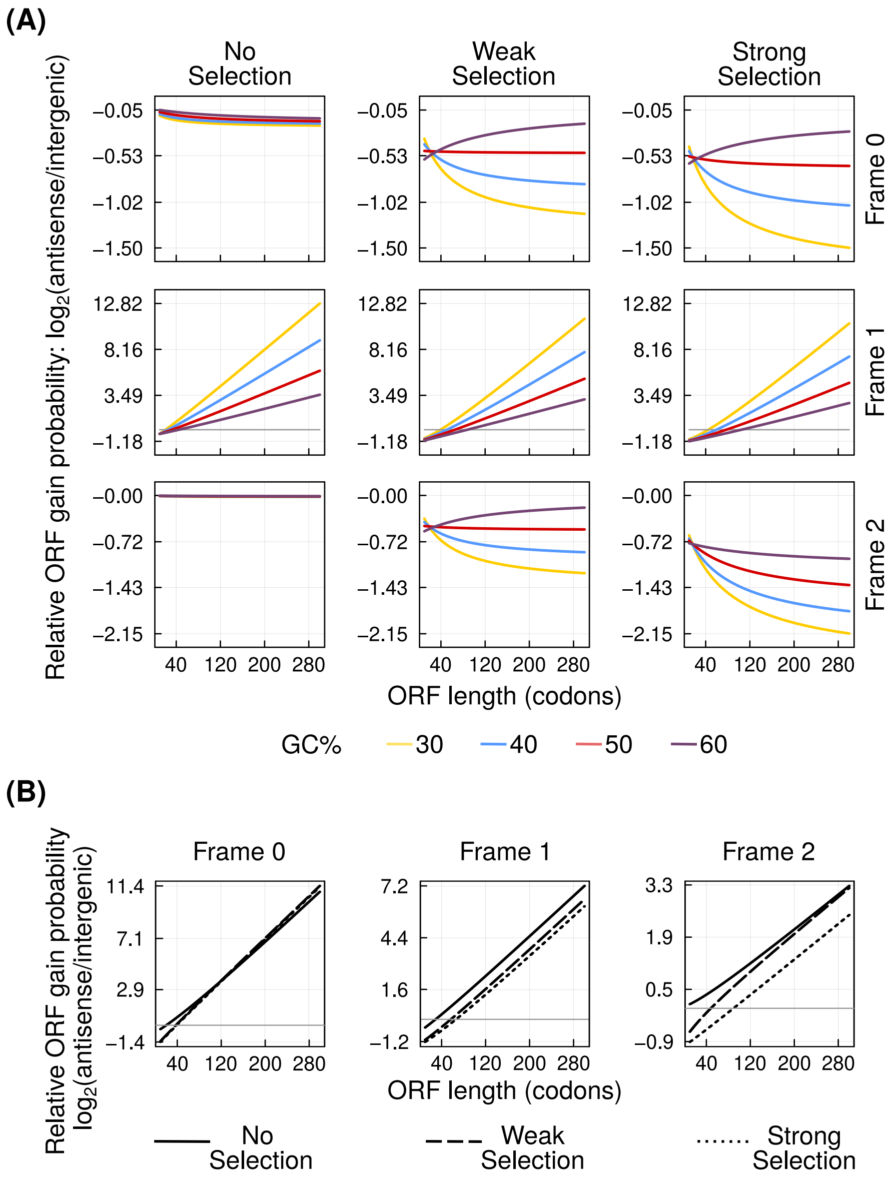

Figure S4: Antisense overlap can facilitate ORF emergence. Panel **(A)** shows the probability of ORF emergence in the three antisense frames (left to right) relative to that in intergenic regions ($\log_2$ ratio, vertical axis), at different intensities of purifying selection (top to bottom). Line colors indicate the GC-content of the ORFs. Panel **(B)** shows the ORFs gain probability in the three antisense frames relative to that in intergenic regions ($\log_2$ ratio, vertical axis), calculated using frequencies of short DNA sequences from *D. melanogaster* genome. Dotted, solid and dashed lines, denote the zero, weak and strong purifying selection, respectively. Horizontal axis in all panels shows the length of the ORFs. For data in both panels, we assume that antisense ORFs overlap completely with the sense ORF.

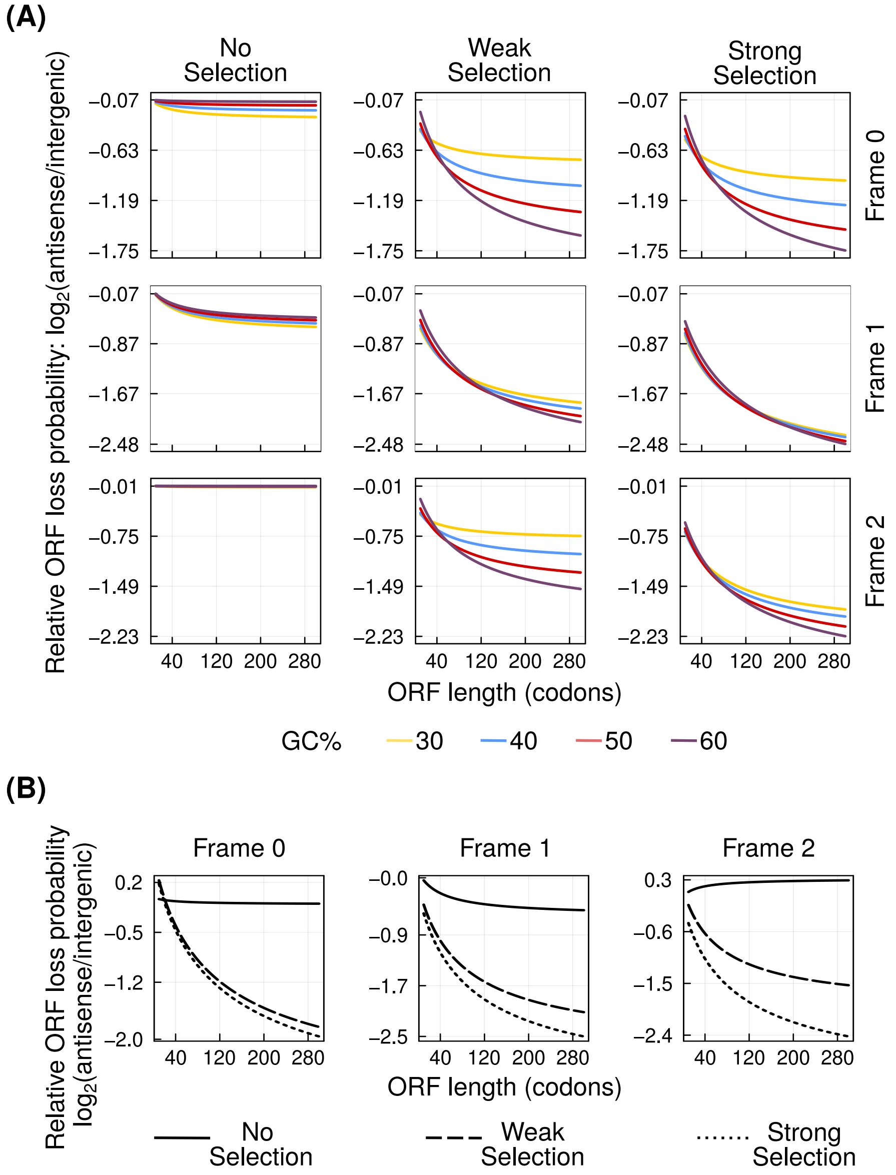

Figure S5: Antisense overlap can reduce ORF loss. Panel **(A)** shows the probability of ORF loss in the three antisense frames (left to right) relative to that in intergenic regions ($\log_2$ ratio, vertical axis), at different intensities of purifying selection (top to bottom). Line colors indicate the GC-content of the ORFs. Panel **(B)** shows the ORFs loss probability in the three antisense frames relative to that in intergenic regions ($\log_2$ ratio, vertical axis), calculated using frequencies of short DNA sequences from *D. melanogaster* genome. Dotted, solid and dashed lines, denote the zero, weak and strong purifying selection, respectively. Horizontal axis in all panels shows the length of the ORFs. For data in both panels, we assume that antisense ORFs overlap completely with the sense ORF.

6.2 Analysis of asORF gain and loss using genomics data

To estimate gain and loss of asORFs we compared their presence or absence in the transcriptome of the different *D. melanogaster* lines. We assume that an ORF emerges only once. That is, if an ORF is detected in five lines, we assume that it emerged once and spread in five lines.

In the first step, we identified ORFs that were shared by several lines. We call defined an

orthogroup as a group of query unique ORF sequences detected in any of the seven lines. Our definition of orthology in this case is very stringent. If an ORF duplicated in two lines, we classified the duplicated copies into two separate orthogroups. That is so because we were interested in the gain and loss of the original ORF and its duplicated copy separately. We also discarded orthogroups where the ORFs from the different lines were not located in the same

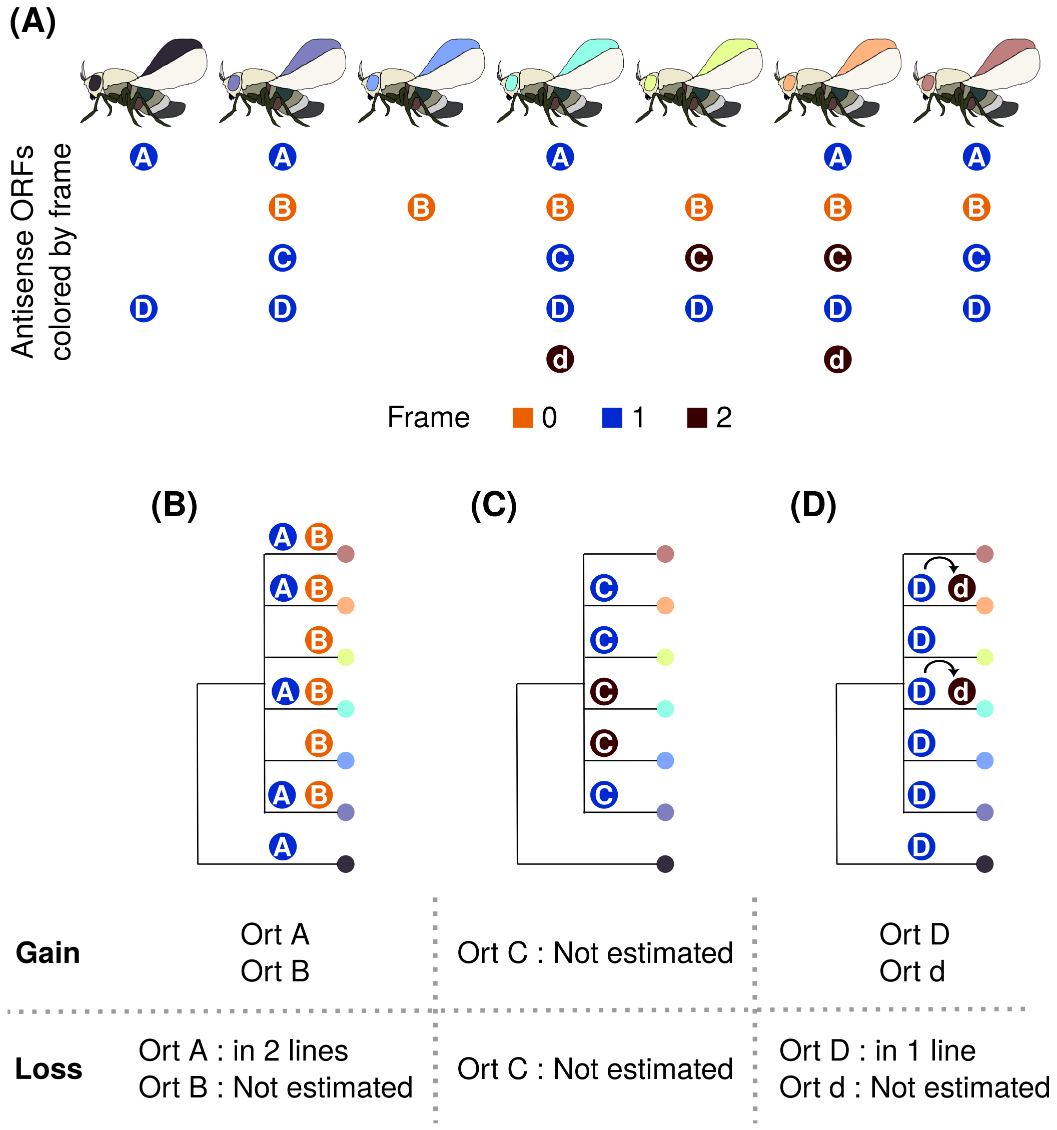

Figure S6: Summary of ORF gain and loss analysis in the seven *D. melanogaster* lines. **(A)** Hypothetical asORF orthogroups denoted by alphabets (A – D) with their frames denoted by the colors orange (0), blue (1) and brown (2). **(B)** The hypothetical example of the orthogroups A and B (containing ORFs A and B, respectively). In both the orthogroups, the ORFs are systematically located in the same frame in every line where they are present. For each of the two orthgroups, we count one gain event. ORF-A is detected in the Zambian outgroup line, but not in the European lines. Thus this ORF is lost in two lines. Because, ORF-B is not detected in the Zambian line, we do not analyse its loss. **(C)** ORF-C is detected in several lines but was located in different frames in the different lines. Thus we do not use this orthogroup for our analysis. **(D)** ORF-D is present in six lines, and has duplicated in two lines (denoted as ORF-D and ORF-d). The duplicated copy (ORF-d) is located in an different frame as ORF-D. Therefore, we classify them into consider 2 orthogroups – the orthogroup containing ORF-D, which is present in the Zambian and some European lines, so that we can estimate its loss. The orthogroup containing ORF-d is only present in two European lines, and therefore we cannot estimate if it was indeed lost in the other lines or only gained in these two lines.

frame. We did so because if would be difficult to infer in which frame (line) the ORF gain occurred first.

To identify orthogroups, we used nucleotide BLAST (Altschul *et al.*, 1990; Camacho *et al.*, 2009). We used nucleotide BLAST instead of protein BLAST for a specific reason – we wanted to identify orthologous asORFs that may be frameshifted. In case of a frameshift, BLASTp may not detect any homology. For the BLASTn analysis, we used an e-value cutoff of 10^{-2} and required a 100% query coverage. Furthermore, we verified that the orthologous asORFs antisense-overlapped with the same protein coding gene. Given these criteria, our algorithm picks the highest scoring hit if there are multiple hits. To keep the analysis focused and less complicated, we only analysed asORF orthologs in which the frame was conserved. Thus our BLAST analyis is overall quite stringent.

Most orthogroups contained only one ORF per line. However, some orthogroups contained several ORFs in a single line, due to tandem duplications. We split these orthogroups such that they contained only one ORF per line, and sorted them according to their frame and the overlapping "sense" ORF. Among the 3536 orthogroups we detected, 105 had several ORFs in several lines. 32 out of these 105 orthogroups contained more than four duplicates in some lines. We discarded these orthogroups because we could not reliably categorize them into sub-orthogroups after splitting them based on frame and position. We also discarded 147 orthogroups were from our analysis because the homologous ORFs were located in different frames.

To estimate the loss, we used the outgroup (Zambian) line. The Zambian populations separated from the European populations between 14000 – 30000 years ago (Li and Stephan, 2006; Laurent *et al.*, 2011). Therefore, if an ORF was found in the outgroup and at least one European line, we assume that it emerged in an ancestral *D. melanogaster* population and was lost in rest of the five European lines. We found 319 orthogroups where the ORF was present in the Zambian line and at least one European line but not all six of them.

7. Effect of mutations on asORFs

In the previous sections, we showed that purifying selection on the sense ORF can affect the emergence and loss of asORFs. We next asked if this purifying selection can also constrain the diversification of the proteins encoded by asORF sequences. To this end, we first calculated the "chemical distance" (δ) between any two amino acids. For this calculation we used a distance matrix that we derived from an experimentally estimated amino acid similarity matrix reported in a previous study (Kim *et al.*, 2009). Next, we calculated the average chemical difference ($\bar{\delta}$) introduced by a random mutation, weighted by the probability of different mutations

(Equation 1). To this end, we created an amino acid distance matrix by modifying the amino acid similarity matrix of Kim *et al.* (2009). Specifically, we subtracted the value of 0.3 from each element of the matrix, reversed the sign of each element, and set the diagonal to zero. By doing this, we set every distance value to be greater than 0. Next, we calculated the average chemical difference introduced by any mutation ($i \rightarrow j$) allowed under a selection regime. Specifically, if i denotes the original codon, j denotes the substituted codon, P_i denotes the probability of finding codon-i, μ_{ij} denotes the probability of codon-i mutating to codon-j, and δ_{ij} denotes the chemical difference between the amino acids encoded by these codons, then the average chemical difference is defined by the following equation:

$$\bar{\delta} = \frac{\sum_i P_i \sum_j \mu_{ij}\delta_{ij}}{\sum_i P_i \sum_j \mu_{ij}} \tag{1}$$

Using $\bar{\delta}$ as a measure of divergence, we estimated the extent to which asORFs in the three frames can diverge as a result of mutations, and due to purifying selection on the sense ORFs. Likewise, we also calculated the divergence of intergenic ORFs as a consequence of random mutations. We found that frame 2 allows maximum divergence of asORFs, under both weak and strong purifying selection on the sense ORF (Figure S7A). asORFs in frame 0 diverge the least. Interestingly, strong selection on sense ORFs increases the divergence of asORFs in frame 2. The reason could be that the few mutations that do occur under strong purifying selection, cause a relatively higher increase in divergence than the more numerous mutations that are allowed to occur under weak purifying selection. We also found that the divergence of asORFs in frame 2 was higher than than that of intergenic ORFs under both selection regimes. We note this result does not mean that intergenic ORFs can diverge less than asORFs. Evolution of intergenic ORFs is not constrained by another DNA sequence. However, as long as the mutants do not affect the organismal fitness, evolution would not be biased towards divergence increasing mutations. Thus random mutations in intergenic ORFs could also consist of many synonymous and chemistry preserving mutations, that are probably disallowed in frame 2 asORFs due to purifying selection on sense ORFs.

In contrast to frame 2, the divergence of asORFs in the other two frames decreased with increasing strength of purifying selection on the sense ORF (Figure S7A). For example, asORFs in frame 0, did not diverge at all when the sense ORF was under strong purifying selection. asORFs in frames 0 and 1 also diverged less than intergenic ORFs under both selection regimes.

We observed identical trends in divergence of asORFs from our analysis based on *D. melanogaster* parameters (Figure S7B).

These findings not negate the fact that intergenic ORFs have less constraints on their evo-

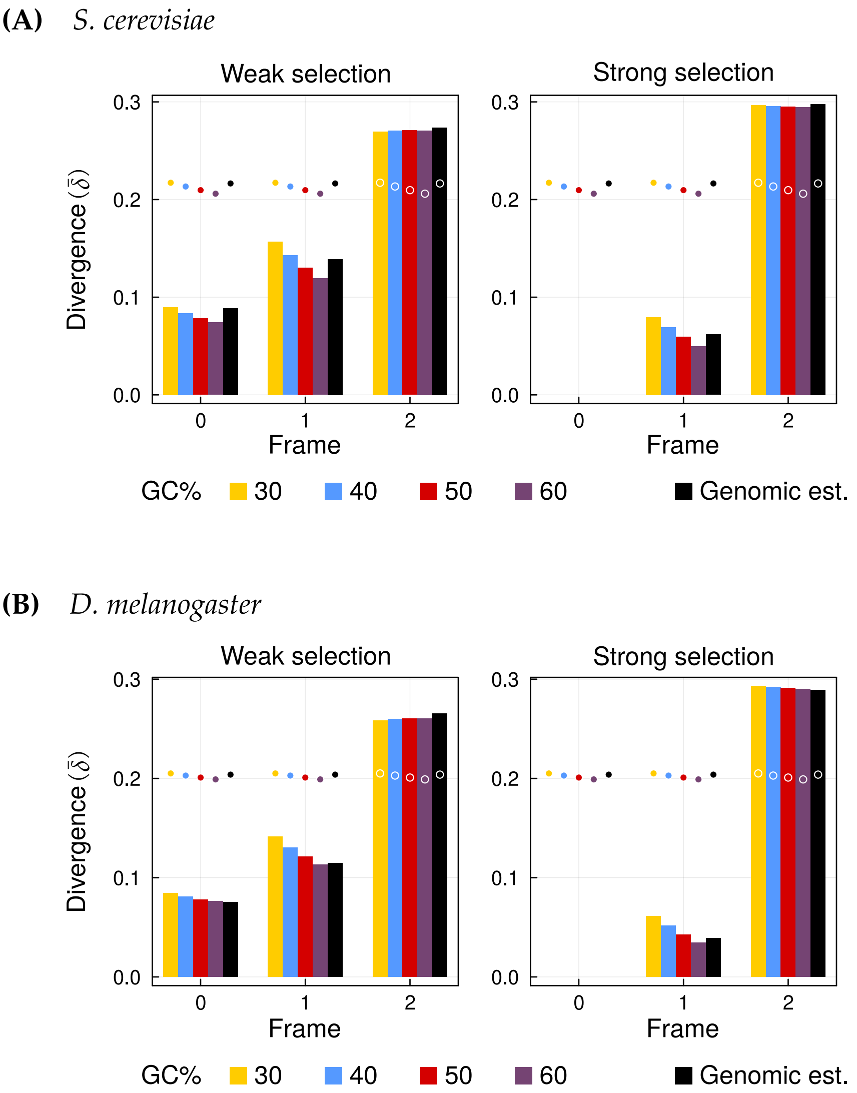

Figure S7: Antisense ORFs in **(A)** *S. cerevisiae* and **(B)** *D. melanogaster*, can diversify when sense ORFs are under purifying selection. Vertical axis denotes the divergence ($\bar{\delta}$) of asORFs due to a random mutation when the sense ORF is under weak (left) or strong (right) purifying selection. Horizontal axes denote the three antisense frames. Colored bars denote divergence values of asORFs with different GC-content, and black bars denote the diversity values calculated using frequencies of short DNA sequences from the yeast genome. Filled circles that are similarly color coded, denote the divergence of intergenic ORFs due to mutations.

lution. Even though chemical consequences of tolerated mutations may be larger in some asORFs than in intergenic ORFs, purifying selection on the sense ORF limits the total number of possible mutations. This would not be the case for intergenic ORFs.

8. Is GC-content a better parameter for asORF probability calculation than global DNA oligomer frequencies?

Any calculation made using an averaged nucleotide composition distribution is likely to be an approximation. It is true for both GC-content (for example, using the average genomic GC-content) or average distribution of DNA oligomers across different genomic loci. Both GC-content and oligomer distribution can be calculated for specific loci, which can make the calculations more realistic. In our plots for of stationary, gain and loss probability based on GC-content (Figures 1B, 3A and 4A), we show four different values of GC-content. They are correct as long as our assumptions hold true. The plots based on DNA oligomer frequencies (Figures 1C, 3B and 4B) may be less realistic because they assume that the oligomer distribution is uniform across the genome (CDS or intergenic regions). Thus the GC-content based plots are more informative.

To understand how realistic averages can be, we performed an empirical analysis of variance of nucleotide composition. Specifically, we normalized the distribution such that the sum of frequencies of a trimer (or GC-fraction) across all loci is equal to one, and calculated the variance of this distribution. We found that GC-content has a smaller variance than that of any DNA trimer (Figure S8). However, this empirical analysis does not prove that GC-content is a better estimate of the real nucleotide distribution.

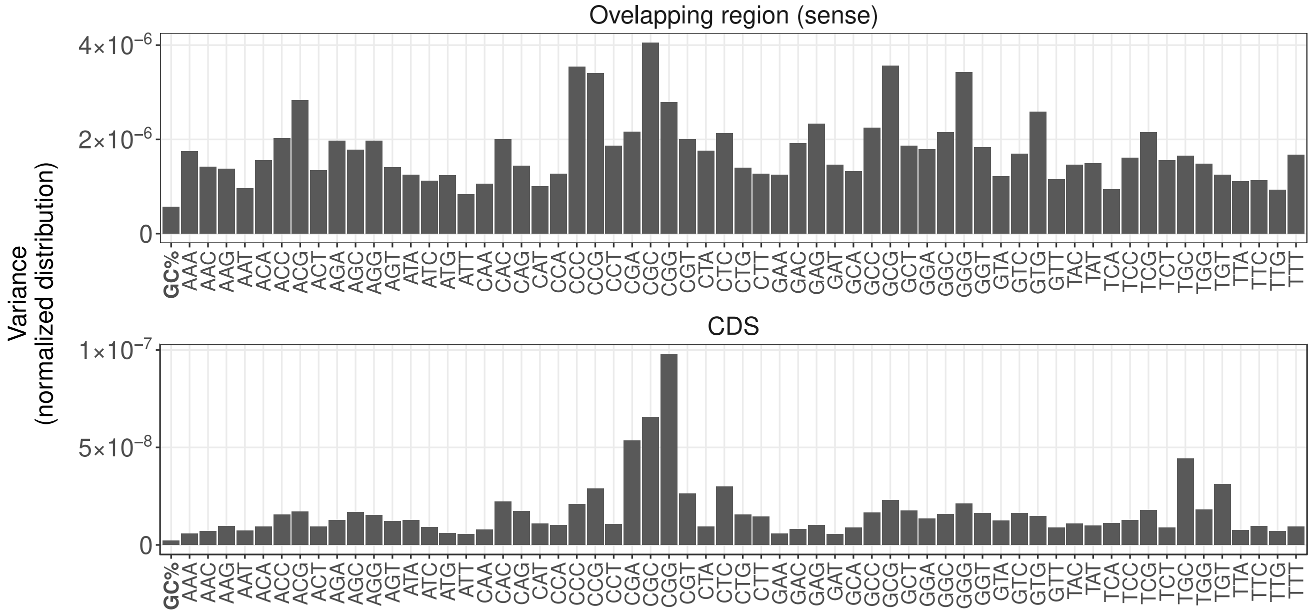

Figure S8: Variance of the normalized distribution of GC-content and of different DNA trimers in *S. cerevisiae*. For coding regions we calculated the frequencies of the different codons as they exist in annotated ORFs (top panel), whereas for regions overlapping with antisense ORFs, we calculated the distribution of DNA trimers using a sliding window (bottom panel). We have excluded stop codons from both the panels.

Ultimately, the most realistic analysis would estimate parameters from each locus separately, and estimate the ORF probabilities specific to that locus. We have indeed done so for calculating expected number of ORFs based on GC-content (main text Table 2). To this end, we calculated the GC-content of each contiguous intergenic or antisense overlapping region, and estimated the ORF probability as well as expected number of ORFs using this specific GC-content. We found that the expected number of ORF using global DNA trimer distribution and locus specific GC-content do not differ significantly.

9. Information on the analysis scripts

All codes and necessary data files are available on GitHub: *BharatRaviIyengar/DeNovoEvolution*

9.1 Modeling and general analysis

Modeling scripts (Julia) are located in the folder "Modeling". *mersfreq.awk* is located in the folder "DataAnalysis".

Script	Description	Input
antisenseGenes.jl	Main model and plotting	Overlapping regions, asORFs (both tabular sequences)
antisenseGenes_supplement.jl	Calculations using oligomer frequencies	Frequencies of trimers, codons and dicodons
nucleotidefuncts.jl	Dependency for basic functions	–
mersfreq.awk	Calculate k-mer frequency	tabular sequence format

9.2 Analysis of *S. cerevisiae* data

These scripts are located in the directory "DataAnalysis/Scerevisiae"

Script	Description	Input
Exp-vs-Obs-asORF.awk	Find expected and observed number of asORFs	Overlapping regions, asORFs (both tabular sequences)
Exp-vs-Obs-igORF.awk	Find expected and observed number of igORFs	Intergenic regions, igORFs (both tabular sequences)
getorf2genomicGFF.awk	Extract GTF from getorf fasta header	getorf output fasta
GTFoverlap2.awk	Find antisense ORFs	GTF of coding regions, asORF GTF

9.3 Analysis of *D. melanogaster* data

The Awk scripts (*.awk) are located in the directory: "DataAnalysis/Dmelanogaster".

The Python scripts (*.py) are located in the directory:
"DataAnalysis/Dmelanogaster/OrthoGroupAnalysis"

Script	Description	Input
antisenseRNA.awk	Find antisense RNAs	Genome GTF
blast2gtf.awk	Convert BLAST tabular output to a GTF	BLAST tabular txt
Exp_vs_Obs_dmel-asORF.awk	Find expected and observed number of asORFs	Overlapping regions, asORFs (both tabular sequences)
Exp_vs_Obs_dmel-igORF.awk	Find expected and observed number of igORFs	Intergenic regions, igORFs (both tabular sequences)
GTFoverlap2.awk	Extracting true antisense ORF coordinates	BLAST output GTF
removespuriousORFs.awk	Remove falsely detected ORFs that lack a stop codon	source RNA fasta, getorf output fasta
extract_sequences.py	Generate asORF fasta (protein + nucleotide)	Genome GTF, asORF GTF
generate_orthogroups.py	Create orthogroups from BLAST hits	BLAST tabular result
filter_orthogroups.py	Filter orthogroups (see section XX)	Orthogroups textfile
analyse_orthogroups.py	Analyse filtered orthogroups for gain and loss	Filtered orthgogroups textfile

References

Acevedo, J. M., Hoermann, B., Schlimbach, T., and Teleman, A. A. 2018. Changes in global translation elongation or initiation rates shape the proteome via the Kozak sequence. *Scientific Reports*, 8(1): 4018.

Altschul, S. F., Gish, W., Miller, W., Myers, E. W., and Lipman, D. J. 1990. Basic local alignment search tool. *Journal of Molecular Biology*, 215(3): 403–410.

Camacho, C., Coulouris, G., Avagyan, V., and others 2009. BLAST+: architecture and applications. *BMC Bioinformatics*, 10(1).

Grandchamp, A., Kühl, L., Lebherz, M., and others 2023. Population genomics reveals mechanisms and dynamics of *de novo* expressed open reading frame emergence in *Drosophila melanogaster*. *Genome Research*, 33(6): 872–890.

Kim, Y., Sidney, J., Pinilla, C., Sette, A., and Peters, B. 2009. Derivation of an amino acid similarity matrix for peptide:MHC binding and its application as a bayesian prior. *BMC Bioinformatics*, 10(1).

Laurent, S. J., Werzner, A., Excoffier, L., and Stephan, W. 2011. Approximate Bayesian Analysis of *Drosophila melanogaster* Polymorphism Data Reveals a Recent Colonization of Southeast Asia. *Molecular Biology and Evolution*, 28(7): 2041–2051.

Li, H. and Stephan, W. 2006. Inferring the Demographic History and Rate of Adaptive Substitution in *Drosophila*. *PLoS Genetics*, 2(10): e166.

Patraquim, P., Magny, E. G., Pueyo, J. I., Platero, A. I., and Couso, J. P. 2022. Translation and natural selection of micropeptides from long non-canonical RNAs. *Nature Communications*, 13(1).

Rice, P., Longden, I., and Bleasby, A. 2000. EMBOSS: The European Molecular Biology Open Software Suite. *Trends in Genetics*, 16(6): 276–277.

Schrider, D. R., Houle, D., Lynch, M., and Hahn, M. W. 2013. Rates and Genomic Consequences of Spontaneous Mutational Events in *Drosophila melanogaster*. *Genetics*, 194(4): 937–954.

Wacholder, A., Parikh, S. B., Coelho, N. C., and others 2023. A vast evolutionarily transient translatome contributes to phenotype and fitness. *Cell Systems*, 14(5): 363–381.e8.

Zhang, Z. and Gerstein, M. 2003. Patterns of nucleotide substitution, insertion and deletion in the human genome inferred from pseudogenes. *Nucleic Acids Research*, 31(18): 5338–5348.
